# Optimizing the design of nanostructures for improved thermal conduction within confined spaces

**DOI:** 10.1186/1556-276X-6-422

**Published:** 2011-06-14

**Authors:** Jianlong Kou, Huiguo Qian, Hangjun Lu, Yang Liu, Yousheng Xu, Fengmin Wu, Jintu Fan

**Affiliations:** 1 College of Mathematics, Physics and Information Engineering, Zhejiang Normal University, Jinhua 321004, PR China; 2 Institute of Textiles and Clothing, The Hong Kong Polytechnic University, Kowloon, Hong Kong, PR China; 3 Department of Mechanical Engineering, The Hong Kong Polytechnic University, Kowloon, Hong Kong, PR China

## Abstract

Maintaining constant temperature is of particular importance to the normal operation of electronic devices. Aiming at the question, this paper proposes an optimum design of nanostructures made of high thermal conductive nanomaterials to provide outstanding heat dissipation from the confined interior (possibly nanosized) to the micro-spaces of electronic devices. The design incorporates a carbon nanocone for conducting heat from the interior to the exterior of a miniature electronic device, with the optimum diameter, *D*_0_, of the nanocone satisfying the relationship: *D_0_^2^*(*x*) ∝ *x*^1/2 ^where *x *is the position along the length direction of the carbon nanocone. Branched structure made of single-walled carbon nanotubes (CNTs) are shown to be particularly suitable for the purpose. It was found that the total thermal resistance of a branched structure reaches a minimum when the diameter ratio, β* satisfies the relationship: β* = *γ*^-0.25*b*^*N*^-1/k*^, where *γ *is ratio of length, *b *= 0.3 to approximately 0.4 on the single-walled CNTs, *b *= 0.6 to approximately 0.8 on the multiwalled CNTs, k* = 2 and *N *is the bifurcation number (*N *= 2, 3, 4 ...). The findings of this research provide a blueprint in designing miniaturized electronic devices with outstanding heat dissipation.

PACS numbers: 44.10.+i, 44.05.+e, 66.70.-f, 61.48.De

## Introduction

With the miniaturization of electronic devices and the increased integration density, the effective dissipation of heat becomes an important requirement for ensuring trouble-free operation [1,2]. The limited space available for heat dissipation, the high energy densities and the dynamically changing, and often unknown, locations of heat sources in micro- and nano-devices [3], make it difficult to apply conventional thermal management strategies and techniques of heat transmission, such as convection-driven heat fins, fluids, heat pastes, and metal wiring [3]. It is a challenge to find the best material and structure for providing excellent heat transfer within the severe space constraints.

Nanomaterials have been widely researched and found to possess novel properties [4-10], for example, single-walled CNTs exhibit extraordinary strength [4], high electrical conductivity (4 × 10^9 ^Acm^-2^) [5] and ultra-high thermal conductivity (3,000 to 6,600 Wm^-2 ^K^-1^) [6,7], which make them potentially useful in many applications in nano-technology, electronics and other fields of material science [11-16]. It therefore follows that nanomaterial should be uniquely suitable for applications requiring exceptional heat transfer properties. Nevertheless, nanomaterials cannot be used directly due to area and volume constraints [17]; particularly in the case of the very small interior of electronic devices which is much smaller than their outside. It is also important to consider the transition from nano- to micro-structure or 'point' to bulk, which occurs from the interior to the exterior of electronic devices. Thus, for example, it is not possible to use single-walled CNTs because of severe space constraints at the interior 'point' level. Therefore, it is necessary to design structures to satisfy space constraints, and, furthermore, to optimize the design to also satisfy the heat conduction requirements.

The use of branched nanostructures has been identified as an effective way to form functional elements that bridge nano- to macro- scale [18-22], for example, actin, cytoskeleton, bone, and collagen fiber networks in biological structures. Recently, Xu and Buehler [22] presented a novel concept involving the use of hierarchical structures as an effective means to create a bridge from the nano- to the macro-scale. Either from the confined interior to the exterior of electronic devices or from nano- to micro-spaces, the space are limited. So, to find the proper structure is necessary. Nevertheless, no work appears to have been done on the optimum design of the heat conduction structures from the confined interior to the exterior of electronic devices and from nano- to micro-spaces.

The objective of the present work is to propose such an optimum design based on the use of carbon nanocones and carbon nanotubes in the form of a conical and branched structure. In the Description of structure section, we give the detailed description of the heat conduction structure, from the interior of an electronic device to micro space, and in the Optimum design section, we present optimum design for heat conduction from the interior to the exterior and nano- to micro-spaces of electronic devices. Lastly, some concluding remarks are given in the Conclusions section.

## Description of structure

**Figure 1:**
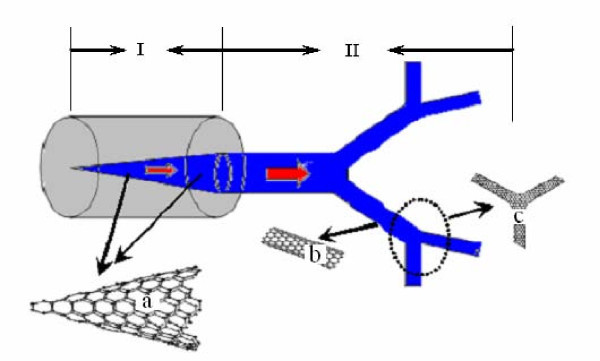
**The design sketch of the total heat conduction structure**. This desgin is from the interior of an electronic device to micro space, which includes two sections: I represents the composite structure of an cylindrical electronic device and an embedded carbon nanocone, the latter being shown in detail in a. II represents the region from the interior to the outside of the electronic device, incorporating the heat conducting branching structure, detailed in b and c. The b and c are single-walled carbon nanotube and branched single-walled carbon nanotube (or single-walled carbon nanotube junction), respectively. The entire branched structure required can be constructed by repeating a finite number of the elements b and c.

**Figure 2:**
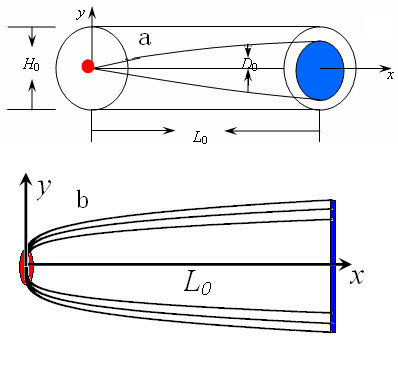
**Sketch of a cylindrical electronic device**. (**a**) a three dimensional sketch of a cylindrical electronic device. The conical section represents the heat conduction medium, the cone showing one of the heat transfer paths from the interior heat source (red) to the edge (blue) of the electronic device, and (**b**) is the cross section optimal designs of the embedded nanocone. Three curves represent the three shapes of the nanocone corresponding to three different volumes of the nanocone (viz. *Vp*).

One promising conductive system which has been designed here, utilizes a carbon nanocone and branched structure consisting of single-walled carbon nanotubes to conduct heat efficiently away from the interior of an electronic device (see Figure 1). The heat conduction route is marked in blue and with red arrows, as shown in Figure 1. It is assumed that the electronic device is cylindrical, and the volumetric heat generation rate from the cylinder is a uniform *q''' *within *V*. A carbon nanocone of ultrahigh thermal conductivity, *k_p _*is inserted into the cylindrical electronic device (or gap) to conduct the heat (See Figure 1(I)). The diameter of the carbon nanocone, *D_0 _*(*x*), (see Figure 2) varies along its length, represented by *x *along the horizontal direction of the carbon nanocone. The heat will be conducted away from the electronic device, and then dissipated into the space through the branches (see Figure 1(II)). The structure is characterized according to each branch as follows: Let the length and diameter of a typical branch at some intermediate level *k *(*k *= 0, 1, 2, 3...*m*, where *m *is total level) be *l_k _*and *d_k_*, respectively, and introduce two scaling factors: *β *= *d*_*k*+1_/*d*_*k *_and *γ *= *l*_*k*+1_/*l*_*k*_, respectively. The elements of the structural design are shown in Figure 1.

## Optimum design

### Interior to the exterior of electronic devices

Because carbon nanocones are so thin and have an ultra-high thermal conductivity, they may be considered as 'one-dimensional', with the heat channeled practically along the *x *direction (i.e., along the axis of the tube). The temperature distribution in the carbon nanocone is shown qualitatively by the red arrows in Figure 1. The structural parameters are detailed in Figure 2(a). The heat generated by the electronic device and entered the carbon nanocone having an ultra-high thermal conductivity *k_p _*is given by , where *H*_0 _is diameter of cylindrical electronic device. The unidirectional heat conduction through the carbon nanocone is given by the following equation [23](1)

The boundary conditions are:(2)(3)

where *L*_0 _is length of cylindrical electronic device or length of embedded nanocone. Applying the boundary condition (2) to Eq. 1 gives:(4)

Integrating Eq. 4 with respect to *x*, the temperature drop from the thin taper end to the thick end of the nanocone can be derived as follows:(5)

In order to achieve maximum heat conduction, *T*_0 _(0) - *T*_0_(*L*_0_) should be minimized. Since the volume of the nanocone:(6)

is confined within a miniaturized device, to minimize *T*_0 _(0) - *T*_0_(*L*_0_) within the given constraints (6), the following integral should be minimized [24]:(7)

where, *λ *is the Lagrange multiplier. The solution of Eq. 7 is the optimal diameter given by . *λ *can be obtained by substituting *D*_0_^2 ^into Eq. 6. We therefore have:(8)

Defining the porosity  and combining Eqs. 8 and 5, gives:(9)

The question now arises as to how good the *D*_0_^2 ^design is relative to that using a uniform path having the thermal conductivity *k_p_*. For the path with a uniformly cylindrical dimension, and porosity , the minimized *T*_0 _(0) - *T*_0_(*L*_0_) can be expressed as follows:(10)

By comparing Eqs. 9 and 10, it can be seen that tapering as represented by Eq. 9, produces a 5.6% lower value for *T*_0 _(0) - *T*_0 _(*L*_0_) than the uniform path design represented by Eq. 10. The optimal designs are illustrated in Figure 2(b). Three curves represent the three shapes of the nanocone corresponding to three different volumes of the nanocone (viz. *Vp*).

### Nano- to micro-spaces

#### Method

As discussed above, optimum heat conduction pathways made of carbon nanocones can be optimally designed to transfer heat efficiently from the interior to the exterior of a miniaturized electronic device; however, heat may still not be rapidly dissipated into the surrounding space as exterior surface of the miniaturized electronic device is small (possibly in nano-scale). It is therefore desirable to channel the heat from the nano-scale exterior surface of the electronic device the micro- or larger space. Bifurcate single-walled CNTs have been produced and exhibited outstanding performance compared to conventional material [25-27]. The idea is inspired by recent work on concept of using a biologically inspired approach of hierarchical structures [22]. The hierarchical structure is an effective way to provide a bridge between the nano- to the macro- level in space. Such structures are considered to be highly advantageous over conventional structures, such as convection-driven heat fins, fluids, heat pastes, and metal wiring, in heat dissipation. However, the optimization of such a branched network of CNTs for heat dissipation has not been analyzed so far. This section thus deals in detail with the optimum design of bifurcate single-walled CNTs for efficiently conducting heat from nano- to micro-spaces.

**Figure 3:**
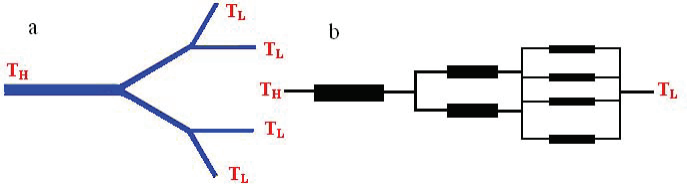
**Schematic diagram of a generalized branched structure**. (**a**) is a schematic diagram of a generalized branched structure of single-walled carbon nanotube with bifurcate number *N *= 2, and total level *m *= 2, which can be considered as an equivalent thermal resistance network to that in (**b**), *T*_H _and *T*_L _representing areas of high and low temperatures, respectively.

Figure 3(a) and 3(b) illustrate a generalized branched structure of single-walled carbon nanotube with bifurcate number *N *= 2 and total level *m *= 2 and the equivalent thermal-electrical analogy network, respectively. According to Fourier's law, the thermal resistance of a single-walled CNT of the *k*th level channel can be expressed as: *R_k _*= *l_k_*/(*λA_k_*) [28], where the [29-31] (The constant *a *is a function of heat capacity, the averaged velocity, mean free path of the energy carriers, temperature, etc. The power exponent *b *= 0.3 to approximately 0.4 [29,30] on the single-walled CNTs, while multiwalled CNTs of *b *= 0.6 to approximately 0.8 [31]). The total thermal resistance, *R_t_*, of the entire branched structure of single-walled carbon nanotubes is given as follows:(11)

where *l*_0 _and *d*_0 _are the length and diameter of the 0th branching level.

Because of space limitations, the branched structure can be equivalent to a single-walled CNT, and with the volume and length being constraints, the design of the branched structure can be optimized. The thermal resistance of the equivalent single-walled CNT, *R_s_*, can be written as:(12)

where: *l_s _*and *A_s _*are the equivalent length (effective length) and cross-sectional area (effective cross-sectional area) of the branched structure, respectively. The branched structure volume, *V*, can be expressed as:(13)

The equivalent length of the branched structure, *l_s_*, is equal to that of the branched structure, *L*, and is given by:(14)

For given an electronic device, the space may be limited by the design. So the length (*L*) of the branched structure may be a limiting factor. With (*L*) being fixed, Eq. (14) implies that, the branched level number *m*, the length (*l_0_*) of the 0th branched single-walled carbon nanotube and the length ratio (*γ*) can be optimized to maximize heat conduction.

According to the relationship between total volume and effective length, i.e., *V = A_s_L*, the effective cross-sectional area, *A_s_*, can be derived as follows:(15)

By substituting Eqs. 14 and 15, into Eq. 12, the thermal resistance, *R_s_*, of the equivalent single-walled carbon nanotube of the same volume as those of the branched structure can be derived as follows:(16)

Combining Eqs. 11 and 16, the dimensionless effective thermal resistance, *R^+^*, of a branched structure is obtained as follows:(17)

*R*^+ ^represents the ratio of the thermal resistance of the branched structure of single-walled carbon nanotubes, *R_t_*, to that of the equivalent *R_s_*, under the constraint of total volume, and which is a function of *γ, β, N, m*, and *b*. As can be seen, equation (17) involves higher order variables, which makes it difficult to attain the optimum scaling relations analytically.

#### Results and discussions

**Figure 4:**
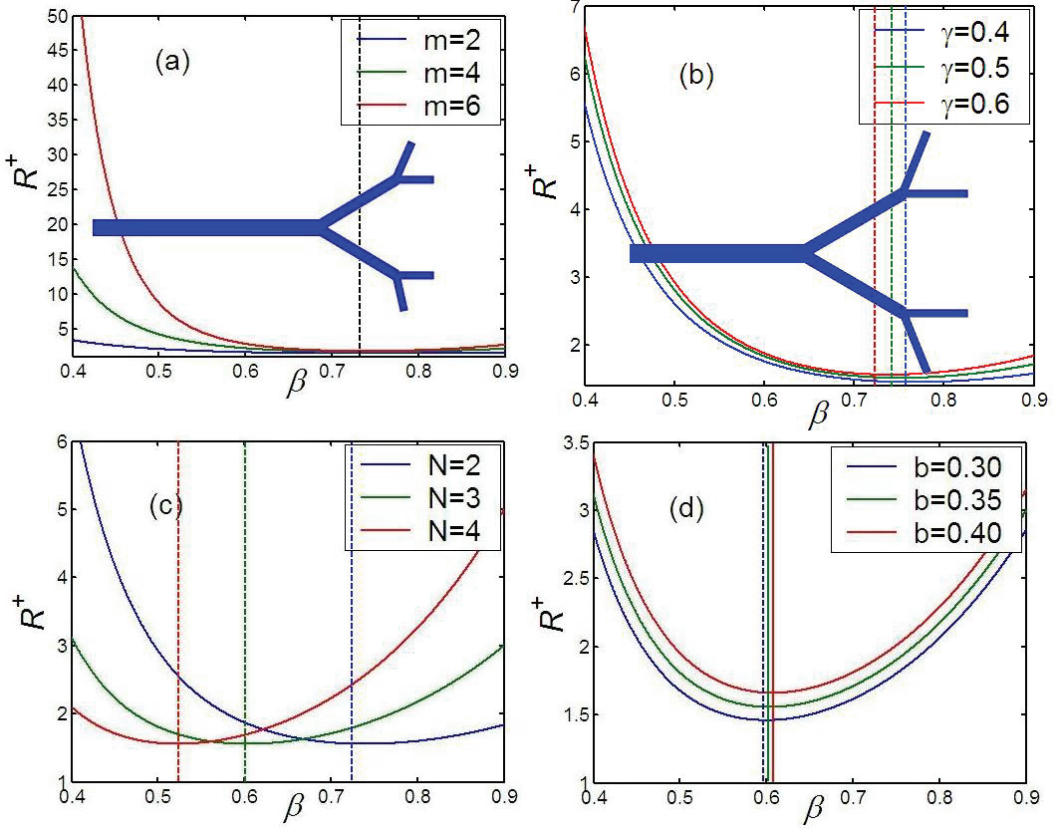
**The effect of structural parameters on effective thermal resistance (*R^+^*)**. (**a**) for different total levels (*m*), with *N *= 2, *γ *= 0.6, and *b *= 0.35, (**b**) for different ratios of length (*γ*), with *N *= 2, *m *= 3, and *b *= 0.35, (**c**) for different bifurcate numbers (*N*), with *m *= 3, *γ *= 0.6, and *b *= 0.35 (**d**) for different power exponents (*b*) with *γ *= 0.6, *N *= 3, and *m *= 3. The optimum design of branched single-wall carbon nanotubes with *m *= 2, *N *= 2 and two different length ratio *γ *are inserted as background in (a) and (b), respectively.

**Figure 5:**
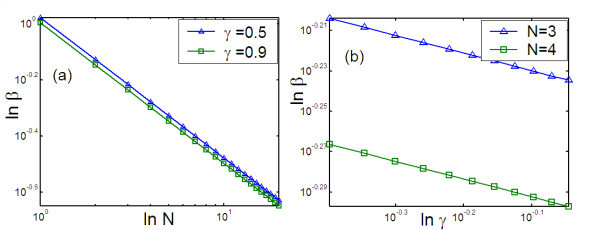
**Scaling relationship of diameter ratio to bifurcate number and rations of length**. Scaling relationship between optimum diameter ratio (*β*) and, (**a**) bifurcate number (*N*) for different ratios of length (*γ*) with *b *= 0.3; (**b**) ratios of length (*γ*) for different bifurcate numbers (*N*) with *b *= 0.35.

To characterize the influence of the structural parameters of branched structures of single-walled carbon nanotubes on the overall thermal resistance, under the volume constraint, the effective thermal resistance of the entire structure (shown in Figure 1(II)) is first analyzed. Based on Eq. 17, the results of the detailed analysis are plotted in Figure 4. Figures 4 shows the effective thermal resistance, *R^+^*, plotted against the diameter ratio *β*, for different values of *m, γ, N*, and *b*, respectively. From these plots, it is apparent that, for a fixed volume, the total branched structure has a higher thermal resistance than the single-walled carbon nanotube. It is therefore strategically important to establish the optimum structure. It can be seen that the effective thermal resistance *R^+^*, first decreases then increases with increasing diameter ratio *β*. There is an optimum diameter ratio β*, at which the total thermal resistance of the branched structure is at its minimum and equal to the thermal resistance of the single-walled carbon nanotube. This represents an optimum condition in designing the branched structure. Furthermore, as can be seen from Figure 4a, the optimum diameter ratio β*, is independent of the number of branching levels *m*. On the other hand, as can be seen from Figure 4b, c, d, length ratio *γ*, the bifurcation number *N*, and power exponents *b *affect the optimum diameter ratio β*. In other words, the value of the optimum diameter ratio β*, depends on the length ratio *γ*, bifurcation number *N *and power exponents *b*. For example, when *b *= 0.3, β* = 0.735 at *N *= 2, and *γ *= 0.6; β* = 0.726 at *N *= 2 and *γ *= 0.7; β* = 0.60 at *N *= 3 and *γ *= 0.6; and β* = 0.593 at *N *= 3 and *γ *= 0.7. In addition, from Figure 4a, it can be seen that the effective thermal resistance *R^+ ^*increases with increase of the number of the branching levels *m*. This is because when the branching levels *m *increases, the network becomes densely filled with much slenderer branches. Figure 4b also denotes that the effective thermal resistance *R^+ ^*increases with the increase of the length ratio *γ*. This is because a higher length ratio *γ *implies longer branches. From Figure 4c, it also can be seen that when the diameter ratio is smaller than optimum diameter ratio (viz., *β *<β*), the effective thermal resistance *R^+ ^*decreases with increase of bifurcation number *N*, while the diameter ratio is bigger than optimum diameter ratio (viz., *β *>β*), the trends is just opposite. The reason is that when *β *<β*, the increase of the parallel channels in every level leads to lower total thermal resistance; but when *β *>β*, the increase of the parallel channels in every level will increase effective volume of total branched structure, leading to an opposite trend. By plotting the logarithm of the optimum diameter ratio β*, against the logarithm of the bifurcation number *N *(see Figure 5), it is apparent that  or β* = *γ*^-0.25*b *^*N*^-1/k*^, where, *γ *is ratio of length, *b *= 0.3 to approximately 0.4 on the single-walled CNTs, *b *= 0.6 to approximately 0.8 on the multiwalled CNTs, *N *is the bifurcation number, *N *= 2, 3, 4,......, k* is the power exponent and *k *= -1/k* = *-*0.5 as shown in Figure 5. From Figures 4c and 5a, it can be observed that there is a smaller optimum diameter ratio with the increase of bifurcation number *N*.

By coupling Eqs. 13 and 14 and applying the optimum diameter ratio, the optimum structural parameters of branched single-wall carbon nanotubes can be derived under the constraint of the total volume (*V*) and length (*L*). The backgrounds of Figure 4a, b show two optimum designs of the branched single-wall carbon nanotubes with *b *= 0.3, *m *= 2, *N *= 2 and different length ratio *γ*. The design in the background of Figure 4a has a smaller value of *γ*, while that of Figure 4b has a greater value of *γ*. To achieve optimum heat conduction and dissipation under the constraints of the total volume (*V*) and length (*L*) of the branched carbon nanotubes structure, the bigger *γ*, the smaller the length (*l_0_*) of the 0th branch.

## Conclusions

In this paper, the optimum design of carbon nanostructure for most efficiently dissipating heat from the confined interior of electronic devices to the micro space is analyzed. It is found that the optimum diameter, *D*_0_, of carbon nanocones satisfies the relationship, . For transmitting heat from the nano-scaled surface of electronic devices to the micro-space, the total thermal resistance of a branched structure reaches a minimum when the diameter ratio, β*, satisfies β* = *γ*^-0.25*b *^*N*^-1/k*^, where, *γ *is ratio of length, *b *= 0.3 to approximately 0.4 on the single-walled CNTS, *b *= 0.6 to approximately 0.8 on the multiwalled CNTS, k* = 2 and *N *= the bifurcation number (*N *= 2, 3, 4,*...*...) under the volume constraints. If space is the only limitation, the optimum diameter remains applicable. These findings help optimize the design of heat conducting media from nano- to micro-structures. It must be noted that the present work is an improvement from the Ref. [22], which showed hierarchical structure is effective in providing a bridge between the nano- to the macro- level for heat transfer. The present work provides a theoretical prediction of how such heat dissipater can be optimally designed.

Despite recent progress in synthesizing and manipulating nanocones and branched single-walled CNTs [25-27,32-34], further work is necessary to perfect techniques and systems for the fabrication of nanostructures and creation of seamless links between the individual single-walled CNT elements of the branched structures, thereby reducing the interfacial thermal resistance [35-37], as well as to precisely control the scale of nanostructures.

## Abbreviations

CNTs: carbon nanotubes.

## Competing interests

The authors declare that they have no competing interests.

## Authors' contributions

JLK performed all the research and drafted the manuscript. HGQ, HJL, YL, and YSX helped to analyze data and contributed equally; WFM and JTF designed the research and supervised all of the studies. All the authors discussed the results and approved the final manuscript.
